# Diagnostics for onchocerciasis in the era of elimination

**DOI:** 10.1093/inthealth/ihx047

**Published:** 2018-02-19

**Authors:** Thomas R Unnasch, Allison Golden, Vitaliano Cama, Paul T Cantey

**Affiliations:** 1 Center for Global Health Infectious Disease Research, College of Public Health, University of South Florida, 3720 Spectrum Blvd., Suite 304, Tampa, FL 33612, USA; 2 PATH, Seattle, WA 98121, USA; 3 Center for Global Health, Centers for Disease Control and Prevention, Atlanta, GA USA; 4 Department of Control of Neglected Tropical Diseases, World Health Organization, Geneva, Switzerland

**Keywords:** Black fly, Diagnostics, Ivermectin, *Onchocerca volvulus*, River blindness

## Abstract

In the past few years, efforts to eliminate onchocerciasis from Africa have intensified. These efforts are primarily based on the mass distribution of the anti-helminthic drug Mectizan™ (ivermectin). This program has led to the development of new guidelines by the World Health Organization for the verification that transmission has been suppressed and eventually eliminated. The requirements of diagnostic tools for this purpose differ in many ways from tests used to diagnose infection in individuals. In this review, we summarize the progress that has been made to identify diagnostics that meet the specialized requirements needed to verify onchocerciasis elimination, discuss why these tests were selected and summarize the needs that still exist to complete the arsenal of diagnostic tools that will be useful as the goal of elimination is achieved.

## Introduction

A recent review has put forth the challenges faced by onchocerciasis elimination programs and how their diagnostic needs evolve as they transition from control verification of elimination.^1^ Here, we will focus upon the diagnostic needs to verify suppression and interruption of transmission in the context of the current WHO guidelines.

Currently, onchocerciasis elimination programs rely primarily on mass drug administration (MDA) of Mectizan™ (ivermectin) to suppress and eventually eliminate transmission of *Onchocerca volvulus*, the causative agent of the disease. The onchocerciasis elimination program strategy is a multi-stage process.^2^ Initially, programs must obtain high enough treatment coverage in the eligible population to stop transmission. Once transmission is suppressed, high coverage must be maintained until all the fertile female parasites either die or become sterile. Once a suitable number of treatments have been given, transmission is believed to have been interrupted. At this point in time, the program conducts a comprehensive survey to demonstrate that transmission has been interrupted. These surveys rely upon measuring parasite exposure in children under 10, who should have remained naive to exposure had transmission been suppressed and whose test results represent a surrogate measure of exposure incidence in the population. The current WHO guidelines state that the upper bound of the 95% confidence interval (CI) for the exposure prevalence in the population of children under 10 cannot exceed 0.1%.^2^

In addition to the measurement of the prevalence of exposure in children, the current guidelines also call for an entomological evaluation to be conducted.^2^ In this evaluation, the upper bound of the 95% CI of the prevalence of flies carrying *O. volvulus* infective larvae must be less than 0.05%.^2^ If both the epidemiological and entomological criteria are met, it may be concluded that transmission has been interrupted. At this point MDA activities can be discontinued, and the program enters the surveillance phase of the elimination process. Three to five years after treatments have been discontinued, the program conducts another entomological evaluation, where once again, the upper bound of the 95% CI of the prevalence of flies carrying *O. volvulus* infective larvae must be less than 0.05%.^2^ If this criterion is met, it can be concluded that onchocerciasis has been eliminated in the evaluation area.

The capability of a diagnostic test is challenged in situations where one is trying to prove that something no longer exists. A test’s sensitivity is the probability of a positive test in a population that is infected, while specificity is the probability of a negative test in an uninfected population. Adjusting test cut-offs to improve one parameter has the opposite effect on the other parameter (e.g., increasing sensitivity will result in a decrease in specificity). Two measures that reflect the accuracy of a test are the positive and negative predictive values. The positive predictive value is the proportion of true positives in the overall number of positives reported by the test, while the negative predictive value is the proportion of true negatives in the overall number of negatives reported. These measures are driven by the sensitivity and specificity of the test and by the infection prevalence in the population. In the setting of a disease elimination program, prevalence is near to or at zero. In the absence of a highly specific test, most positive results will be false positives, and the positive predictive value of the test will be very low.

Because the elimination strategy requires that all eligible individuals in a targeted community regardless of infection status be treated, the results of any particular test will not affect an individual’s treatment. Thus, test sensitivity can be sacrificed to maximize specificity and minimize the number of false positive results in a situation where one is interested in population prevalence, as the number of people sampled can be increased to compensate for decreases in sensitivity.^3^ For example, as discussed above, the epidemiological criterion for stopping MDA in the current WHO guidelines for verifying interruption of transmission of *O. volvulus* requires testing enough children to conclude that the upper bound of the 95% CI of the prevalence of exposure in at-risk children less than 10 years old is less than 0.1%.^2^ Assuming a test with 100% sensitivity and 100% specificity, 3000 children must be tested and have negative results to meet this criterion.^4^ The sample size needed for a test exhibiting less than 100% sensitivity is roughly the sample size needed for an assay with 100% sensitivity divided by actual sensitivity.^3^ For example, for an assay with 70% sensitivity, one needs to test approximately 4285 individuals to meet the WHO criterion. In contrast to sensitivity, the specificity of an assay sets a floor of a prevalence value below which it is very difficult to measure. For example, if we employ an assay with a specificity of 99%, roughly 1% of the samples will test falsely positive in every trial. This means that the test on average will report a prevalence rate of 1%, even if the true prevalence is 0%. To detect a true prevalence of 0.1% in this case, sufficient individuals must be tested to ensure that a result reporting a 1.1% prevalence is significantly different from the 1% false positive rate. To achieve this goal would require testing over 63 000 individuals (C. R. Katholi, personal communication). Thus, ensuring a very high degree of specificity in the assay used to verify transmission elimination is paramount.

## Detection of parasite presence in humans

Traditionally, microscopic examination of skin biopsies (snips) has been the gold standard for diagnosis and surveillance of *O. volvulus* infection.^5^ Snips generally exhibit a high degree of specificity, as *O. volvulus* and *Mansonella streptocerca* are the only filarial parasites in onchocerciasis endemic areas whose larvae inhabit the skin of an infected individual, and the larvae of these two species are readily distinguishable. However, snips are generally insensitive indicators of infection and the sensitivity of the skin snip decreases as the density of microfilaria in the skin decreases.^6^ This problem is exacerbated in populations that have received MDA with Mectizan™, which is a potent microfilaricide that effectively reduces microfilarial density in communities under successful MDA. A recent report suggests that the sensitivity of the conventional skin snip assay when compared with PCR in areas subject to successful MDA ranged from 76% in Uganda to 29% in Ethiopia.^6^

Attempts have been made to increase the sensitivity of the skin snip assay by replacing the microscopic examination of the snip with detection of amplified parasite DNA. The original DNA amplification assay for *O. volvulus* targeted a tandemly repeated sequence present in the *O. volvulus* genome with a unit length of roughly 150 bp, designated the O-150 repeat.^7^ This repeat family was found to be present in other species of the genus *Onchocerca*, but was lacking in the other human filarial parasites.^7^ The repeat family consisted of genus, species and strain-specific repeat units,^8^ permitting the development of species and strain- specific probes that could be used to classify the amplicons generated from amplification of the O-150 repeat.^9^ Real-time PCR^6,10^ and isothermal loop amplification (LAMP)^11,12^ assays have also been developed for the amplification of *O. volvulus* DNA, decreasing the limit of detection of these assays to significantly less than a single parasite and permitting rapid colorimetric detection of the amplified products.^13^ Most of these assays have targeted the O-150 repeat, though similar assays have been reported that target moderately repeated DNA sequences (rRNA genes,^6^ mitochondrial genes^11^) or even single copy genes.^13^ As might be predicted, using a DNA amplification assay to detect the presence of parasite DNA rather than using microscopy to detect the parasite itself has generally been found to increase the sensitivity of the skin snip.^6,10,14^ As a result, amplification of *O. volvulus* parasite DNA from skin snips has become the accepted standard for the diagnosis of patent *O. volvulus* infection in humans.^2^

Despite the high specificity exhibited by the skin snip assays, these are not generally applicable for demonstration of transmission interruption for several reasons. First, Mectizan™ rapidly reduces the number of microfilariae in the skin to zero or near zero,^15^ reducing the positive predictive value of the assay. Second, obtaining the biopsies is both painful and carries some risk of transmitting blood-borne infections, leading to community resistance.^16^ Together, these drawbacks led WHO to recommend against the use of skin snip-based assays as a primary diagnostic for the verification of elimination.^2^

An alternative method to skin snipping is the diethylcarbamazine (DEC) patch test. This test is based upon the observation that application of the anti-helminthic DEC to the skin of microfiladermic individuals infected with *O. volvulus* elicits a localized rash within 24–48 h.^17^ This is less invasive than collecting skin biopsies and thus potentially more acceptable to communities. DEC patch performance has varied widely in the different trials, with sensitivities ranging from 36 to 83% depending upon the comparator test (skin snip microscopy or PCR) used as the gold standard.^18–20^ Specificities were generally not high enough for the DEC patch to be used as a stand-alone test in areas with low prevalence.^19^ Furthermore, the sensitivity and specificity of this test was not evaluated in situations where successful MDA programs were ongoing. These issues have prevented the DEC patch test from being recommended by WHO for the verification of elimination.^2^

## Serological tests to detect exposure to *O. volvulus*

Preliminary studies employing low molecular weight (LMW) *O. volvulus* protein fractions as antigens in serological assays resulted in promising levels of sensitivity and specificity.^21,22^ As a result, multiple LMW antigens were produced and evaluated for the serodiagnosis of onchocerciasis (Table 1).

**Table 1.** Candidate antigens considered for diagnosis of onchocerciasis

MW (kDa)	Antigen(s)	Associated protein	Specificity (%)	Sensitivity (%)	Reference	Test used
15	OV103	MF surface associated protein	70	57	23	ELISA
	Ov-MSA-1		99	89	24	IgG4 LIPS assay
16	OV16	Phosphatidyl ethanolamine binding protein	96	96	25	ELISA
17	OV10	Cysteine proteinase inhibitor	100	61	26	ELISA
	OC 9.3		83	68	26	ELISA
	OC 9.3		100	84	27	ELISA
	OV-CPI		99	32	24	IgG4 LIPS assay
	OV7		n/a	75	28	ELISA
19–20	OvMPB/10	Not determined	100	78	29	ELISA
20	OV11	Retinol binding protein	96	54	26	ELISA
	OvMPB/11		99	65	29	ELISA
	Ov-Far-1		100	100	24	IgG4 LIPS assay
	OV20/36M		100	45	30	ELISA
	OV20/OVS1		81/85	75/89	31	ELISA
20–23	OV 31	Not determined	92	68	26	ELISA
	OV 31			100	30	Microplaque spot analysis
	OV22/31M		100	74	30	ELISA
28	MSP-2	Major sperm protein	85	100	32	Dot blot assay
33	OC 3.6	Aspartyl protease inhibitor	n/a	93	27	ELISA
	OV33-GST		96	93	33	ELISA
	C27		n/a	82	34	Recombinant OV33/ELISA
	C71		n/a	85	34	Recombinant OV33/ELISA
	Ov-API-1		100	100	24	IgG4 LIPS assay
	OV 33/5M		100	n/a	30	ELISA

Currently, the field has settled upon assays detecting antibodies against the Ov16 antigen for monitoring exposure to *O. volvulus*. This antigen is present in all lifecycle stages^35^ and elicits detectable antibody responses prior to the appearance of microfilaria in some chimpanzees experimentally infected with *O. volvulus*^36^ and in some children exposed to the parasite in endemic communities.^37^ While the initial assessment of the utility of the Ov16 antigen was assessed using detection of total IgG against Ov16, the IgG4 subtype response was the most specific.^38,39^ This is perhaps not surprising, as measurements of IgG isotypes in filarial infections revealed that IgG4 accounts for up to 95% of the IgG response to these infections.^40^ All current versions of the Ov16 assay have focused on IgG4 detection; however, the IgG4 response takes time to develop^41^ and thus will not immediately reflect exposure to *O. volvulus*. The Ov16 ELISA is now recommended by WHO guidelines for demonstrating the interruption of transmission of *O. volvulus*.^2^ Most Ov16 ELISA methods utilize dried blood spots (DBSs) as the input sample type. DBS samples are relatively stable, and can be easily collected and transported to a central facility for testing later. The cost of the reagents alone required for processing a single sample (generally run in duplicate) are approximately US $0.30, and one individual can process roughly 15 000 samples per year. However, this estimate does not include the cost of shipping to endemic laboratories. Fully burdened costs for running Ov16 ELISA are thus location-specific and should be analyzed as part of efforts to improve laboratory capacity in countries utilizing serological surveillance.

The Ov16 antigen was adapted into a rapid format card test by AMRAD ICT (Australia) with a reported sensitivity of 90.6%.^42^ Despite promising field performance, production of this card test ceased in the year 2000. Recently, the interest in an Ov16 rapid test was revived, spurring the development of two rapid diagnostic tests (RDTs) incorporating the Ov16 antigen which are now commercially available. These consist of a single IgG4 rapid test and a combination test utilizing Ov16 and the *W. bancrofti* antigen Wb123.^43,44^ The product inserts report the sensitivity of the Ov16 single test to be 81.1% and the Ov16 test line of the biplex test to be 81.33%, respectively.^45^ The specificity is listed at 99.0% for the single Ov16 test and 100% for the biplex test, although it was reported to be 1–2% lower than these values in published studies using early prototypes.^43,44^ As part of the development of the RDT, a recombinant human IgG4 positive control antibody specific for Ov16 was also developed,^46^ providing a highly pure, consistent and long-term source of positive control for both the ELISA and RDT assays.

The new Ov16-containing RDTs for anti-Ov16 serology are rapidly being incorporated into field studies and surveillance activities. While feasibility and acceptability of the rapid tests in surveillance have been demonstrated,^47^ field-based studies that include performance data using the commercial product are still pending publication. Newly released WHO guidelines recommend evaluation of the performance of these RDTs prior to their use in stop-MDA assessments;^2^ hopefully, data to assess the utility of the Ov16 RDT in the verification of elimination will be available soon.

## Entomological surveillance of *O. volvulus* transmission

The transmission cycle of *O. volvulus* includes blackfly vectors of the genus *Simulium*. The most direct measure of the status of transmission is to measure infectivity in the black fly vector population itself. Entomological surveillance has the advantage that it eliminates the time lag inherent in the assays focusing on the human host population, where detectable patency lags infection by 12–18 months.^37^ The disadvantage to entomological surveillance is that large numbers of vector insects must be caught and tested. The current WHO guidelines call for testing sufficient numbers of flies to ensure that the upper bound of the 95% CI of the prevalence of flies carrying infective larvae is less than 0.05% (1/2000).^2^ Meeting this criterion requires testing at least 6000 flies and having all test negative to meet this criterion.^4^ Annual transmission potential can be used as an alternative criterion when it is not possible to capture 6000 flies due to low prevalence of flies.^2^

The traditional method of determining the prevalence of flies carrying infective larvae has been through field dissection of captured flies. However, this method suffers from two disadvantages. First, dissection is expensive because it requires a trained entomologist, a field microscope and a lot of time. Second, a more significant disadvantage is that *Simulium damnosum* sensu *lato*, the major vector of *O. volvulus* in Africa, also serves as the vector for zoonotic species of *Onchocerca* that do not infect humans.^48^ The larvae of these animal parasites are difficult or impossible to distinguish morphologically from *O. volvulus*; thus, dissection data can result in overestimates of the intensity of transmission.

A solution to the inability of vector dissection to accurately describe transmission was to develop a specific PCR, and techniques to overcome the cost and time implications of having to test 6000 blackflies individually. Although several DNA amplification assays have been developed, the O-150 PCR has been the assay that has been used most widely.^13,49^ This PCR method distinguishes *O. volvulus* from other Onchocerca present in *S. damnosum *s.l.**, thereby improving the accuracy of the transmission estimates.^9^ Screening efficiency using the PCR is superior to dissection, as the PCR assay can be applied to screening pools of flies.^49^ However, one potential drawback to screening pools of flies is that the O-150 PCR is not quantitative and inhibitors present in the DNA preparations can reduce the efficiency of the PCR, making it impossible to get accurate estimates of the number of parasites present in a positive pool, even when the O-150 PCR is adapted to a quantitative PCR format. Thus, it is not possible to determine whether a positive pool contains a single infectious fly or multiple infectious flies. This problem was overcome by applying probability distribution estimates when it was realized that although it was not possible to determine how many positive flies were in a positive pool, it was possible to say with certainty that negative pools contain no infectious flies. If the infectious flies are randomly distributed among the collection (something easily accomplished when arranging the flies into pools with the maximum number of flies appropriate for processing), it is possible to use a probability distribution to calculate the probability estimate of the number of infected flies in a pool, given the proportion of negative and positive pools and the number of flies contained in each pool.^49^ The mathematics behind this observation were incorporated into a program (PoolScreen) that calculates the prevalence of infectious flies and associated confidence intervals from the proportion of positive pools, the pool size and the number of pools screened. Field studies conducted in both Africa^50^ and Latin America^51^ validated this approach. The O-150 PCR has subsequently been widely applied to collect entomological data verifying elimination of transmission of *O. volvulus* in Mexico,^52^ Guatemala^53^ and one focus in Sudan.^54^

PCR pool-screening techniques have overcome most of the operational difficulties associated with meeting the WHO guideline’s vector criterion. Screening the 6000 flies necessary to meet this requirement by screening just 60 pools of 100 flies each would take one individual less than 1 week. Pool screening also dramatically reduces the cost and time necessary to process the samples, when compared to analyzing each insect individually. The cost of reagents to process a single pool of 100 flies roughly US$6.90 per pool, or roughly US$ 0.07 per insect. A single individual can process roughly 4000 pools or 400 000 individual insects in a year. Furthermore, the collected insects can be stored in alcohol indefinitely at room temperature, minimizing the logistical difficulties encountered when shipping the collections to a central laboratory for analysis.

Collecting the necessary number of flies now represents the main challenge to implementing the entomological surveys. Currently, the standard method of collecting vector black flies is human landing collections (HLCs), which are quite inefficient, as a team of two collectors can only collect one person-day's worth of flies per day. However, recent reports suggest that a new trap platform, known as the Esperanza Window Trap (EWT), may represent an effective alternative to HLCs for collecting vector black flies.^55^ Studies in Mexico demonstrated that EWTs operated by residents of the affected communities could collect sufficient numbers of flies to certify these communities were free of *O. volvulus* transmission.^56^ If the EWT platform has an equivalent performance in Africa when operated by community members, it may overcome the difficulty in obtaining sufficient numbers of vector flies to demonstrate suppression and interruption of transmission.

## Conclusion and future directions

Although the current diagnostic tools have served well for verifying suppression and interruption of transmission of *O. volvulus* in most countries in Latin America and in several foci in Africa, there are several tools that could accelerate program activities targeting the elimination of onchocerciasis. One of the most pressing needs is to define the sampling schemes for verification of elimination in Africa. In Latin America, the approach that was used by the Onchocerciasis Elimination Program for the Americas was to identify sentinel hyper-endemic communities in each focus prior to the beginning of the program.^57^ The epidemiological and entomological indicators described previously were then used to follow the decline and eventual elimination of transmission in these sentinels. In general, the foci in Latin America were also isolated from one another, making the definition of a focus a simple matter. In contrast, sentinel communities were often not identified prior to the start of onchocerciasis control in Africa. Furthermore, the foci that exist in Africa are often not isolated, with the potential for reintroduction of the parasite either through migration of infected people or by wind-borne flies.^58^ It is thus necessary to gain a better understanding of what the limits to a focus are and, once this is done, how these foci should be sampled. For example, the epidemiological metrics needed for stopping MDA still need to be clarified, including how many communities should be enrolled to obtain reliable data on transmission in a focus, how many people in each community should be sampled and how the communities to be included should be chosen. Similarly, for the entomological metrics, it is not clear how many different sites should be included, how many flies need to be collected from each site, or for how long and how often the collections should be carried out.

A second need is for the current seroprevalence cut-offs in the 2016 WHO guidelines to be re-evaluated in light of the recent progress in modeling the transmission of *O. volvulus*. For example, a recent study has suggested that the prevalence of exposure in children that would indicate the parasite population is irreversibly headed to extinction may often be higher than 0.1%, though this is dependent on the baseline endemicity.^59^ A similar re-evaluation of the entomological metrics is also in order, perhaps with more focus on the annual transmission potential, rather than simple prevalence of infection. Once these analyses are completed, they should be used in conjunction with methods to both more accurately determine test performance in relevant settings and calculate sample sizes that take the sensitivity and specificity of the tests to be employed into account.^3^

Specificity is paramount when choosing tests for verifying elimination. However, a single-antigen antibody test often cannot deliver a very high degree of specificity without suffering a dramatic loss of sensitivity. One solution is to use a confirmatory test that is independent from the primary test, and require that both the primary and confirmatory tests be positive before declaring a sample positive. Incorporating a second parasite antigen marker in an RDT, with a distinct line for each marker, could allow tailoring of the test to provide either highest sensitivity (requirement of only a single test line present to be positive) or highest specificity (both test lines must be positive). In a similar vein, while the sensitivity and specificity of the PCR assays used in black flies generally approach 100%, a major technical obstacle with these assays is the potential for amplicon contamination resulting in false positive signals. Including an independent PCR assay targeting a second genomic sequence is one way to overcome this problem. Given that there are other antigens already available that can be used to detect exposure to *O. volvulus*, and other PCR targets already identified for detection of parasite DNA, studies and necessary product development should be undertaken to determine which combination of tests would result in the highest combined sensitivity and specificity for verifying elimination.

Finally, the available diagnostics do not directly detect potentially fertile female parasites in the human population that could restart reproduction once MDA is stopped and which may pose a risk for recrudescence. Even though not all adult worms need to be sterile or dead for transmission to be irreversibly interrupted, it would be beneficial to have a method to detect and treat people who harbor adult worm infections. The detection of adult worm infections could allow targeted treatment with medications that can permanently sterilize or kill the worms, either to accelerate program progress to achieving interruption of transmission or to further lower the probability of recrudescence of transmission in mop-up operations that occur after MDA has been stopped but transient transmission is detected. Safe treatments are available that can permanently sterilize and eventually kill the adult females,^60^ so the missing piece is the diagnostic test to identify people infected with fecund adult females. Some progress has been made in developing assays to detect viable adult parasites in humans. These include specific metabolites produced by female worms^61,62^ and detection of parasite miRNA in the blood of infected individuals.^63^ Assays that could detect patent infections in the face of an effective MDA program have great potential for both speeding up the process of elimination and ensuring that the infection does not recrudesce once MDA is withdrawn.

## References

[1] Vlaminck J, Fischer PU, Weil GJ Diagnostic tools for onchocerciasis elimination programs. Trends Parasitol 2015;31(11):571–82. 26458784 10.1016/j.pt.2015.06.007

[2] World Health Organization Guidelines for stopping mass drug administration and verifying elimination of human onchocerciasis: Criteria and Procedures. Document # WHO/HTM/NTD/PCT/2016.1; Geneva: WHO Press; 2016. 26913317

[3] Johnson WO, Su C-L, Gardner IA et al Sample size calculations for surveys to substantiate freedom of populations from infectious agents. Biometrics 2004;60(1):165–71. 15032786 10.1111/j.0006-341X.2004.00143.x

[4] Basanez MG, Rodriguez-Perez MA, Reyes-Villanueva F et al Determination of sample sizes for the estimation of *Onchocerca volvulus* (Filarioidea: Onchocercidae) infection rates in biting populations of *Simulium ochraceum* s.l. (Diptera: Simuliidae) and its application to ivermectin control programs. J Med Entomol 1998;35(5):745–57. 9775604 10.1093/jmedent/35.5.745

[5] Kale OO, Bammeke AO, Ayeni O An evaluation of skin snip techniques used in the quantitative assessment of microfilarial densities of Onchocerca volvulus. Bull World Health Organ 1974;51(5):547–9. 4549504 PMC2366323

[6] Thiele EA, Cama VA, Lakwo T et al Detection of *Onchocerca volvulus* in skin snips by microscopy and real-time polymerase chain reaction: implications for monitoring and evaluation activities. Am J Trop Med Hyg 2016;94(4):906–11. 26880774 10.4269/ajtmh.15-0695 PMC4824238

[7] Meredith SEO, Lando G, Gbakima AA et al *Onchocerca volvulus*: application of the polymerase chain reaction to identification and strain differentiation of the parasite. Exp Parasitol 1991;73:335–44. 1915748 10.1016/0014-4894(91)90105-6

[8] Zimmerman PA, Toe L, Unnasch TR Design of Onchocerca DNA probes based upon analysis of a repeated sequence family. Mol Biochem Parasitol 1993;58:259–69. 8479450 10.1016/0166-6851(93)90047-2

[9] Toe L, Merriweather A, Unnasch TR DNA probe based classification of *Simulium damnosum* s.l. borne and human derived filarial parasites in the Onchocerciasis Control Programme area. Am J Trop Med Hyg 1994;51:676–83. 7985761

[10] Lloyd MM, Gilbert R, Taha NT et al Conventional parasitology and DNA-based diagnostic methods for onchocerciasis elimination programmes. Acta Trop 2015;146:114–8. 25818324 10.1016/j.actatropica.2015.03.019

[11] Lagatie O, Merino M, Batsa Debrah L et al An isothermal DNA amplification method for detection of *Onchocerca volvulus* infection in skin biopsies. Parasit Vectors 2016;9(1):624. 27906100 10.1186/s13071-016-1913-7 PMC5134125

[12] Alhassan A, Osei-Atweneboana MY, Kyeremeh KF et al Comparison of a new visual isothermal nucleic acid amplification test with PCR and skin snip analysis for diagnosis of onchocerciasis in humans. Mol Biochem Parasitol 2016;210(1–2):10–12. 27473357 10.1016/j.molbiopara.2016.07.006

[13] Poole CB, Li Z, Alhassan A et al Colorimetric tests for diagnosis of filarial infection and vector surveillance using non-instrumented nucleic acid loop-mediated isothermal amplification (NINA-LAMP). PLoS One 2017;12(2):e0169011. 28199317 10.1371/journal.pone.0169011 PMC5310896

[14] Zimmerman PA, Guderian RH, Aruajo E et al Polymerase chain reaction-based diagnosis of *Onchocerca volvulus* infection: improved detection of patients with onchocerciasis. J Infect Dis 1994;169(3):686–689. 8158053 10.1093/infdis/169.3.686

[15] Remme J, Baker RH, DS G et al A community trial of ivermectin in the onchocerciasis focus of Asubende, Ghana. I. Effect on the microfilarial reservoir and the transmission of *Onchocerca volvulus*. Trop Med Parasitol 1989;40:367–74. 2617046

[16] Diawara L, Traore MO, Badji A et al Feasibility of onchocerciasis elimination with ivermectin treatment in endemic foci in Africa: first evidence from studies in Mali and Senegal. PLoS Negl Trop Dis 2009;3(7):e497. 19621091 10.1371/journal.pntd.0000497 PMC2710500

[17] Kilian HD The use of a topical Mazzotti test in the diagnosis of onchocerciasis. Trop Med Parasitol 1988;39:235–8. 3194667

[18] Ozoh G, Boussinesq M, Bissek AC et al Evaluation of the diethylcarbamazine patch to evaluate onchocerciasis endemicity in Central Africa. Trop Med Int Health 2007;12(1):123–9. 17207156 10.1111/j.1365-3156.2006.01750.x

[19] Boatin BA, Toe L, Alley ES et al Detection of *Onchocerca volvulus* infection in low prevalence areas: a comparison of three diagnostic methods. Parasitology 2002;125(Pt 6):545–52. 12553573

[20] Toe L, Adjami AG, Boatin BA et al Topical application of diethylcarbamazine to detect onchocerciasis recrudescence in west Africa. Trans R Soc Trop Med Hyg 2000;94(5):519–25. 11132381 10.1016/s0035-9203(00)90073-7

[21] Lucius R, Buttner DW, Kirsten C et al A study on antigen recognition by onchocerciasis patients with different clinical forms of disease. Parasitology 1986;92 (Pt 3):569–80. 3526262 10.1017/s0031182000065458

[22] Cabrera Z, Parkhouse RM, Forsyth K et al Specific detection of human antibodies to *Onchocerca volvulus*. Trop Med Parasitol 1989;40(4):454–9. 2696081

[23] Lustigman S, Brotman B, Johnson EH et al Identification and characterization of an *Onchocerca volvulus* cDNA clone encoding a microfilarial surface-associated antigen. Mol Biochem Parasitol 1992;50:79–94. 1542318 10.1016/0166-6851(92)90246-g

[24] Burbelo PD, Leahy HP, Iadarola MJ et al A four-antigen mixture for rapid assessment of *Onchocerca volvulus* infection. PLoS Negl Trop Dis 2009;3(5):e438. 19436728 10.1371/journal.pntd.0000438 PMC2677158

[25] Lobos E, Altmann M, Mengod G et al Identification of an *Onchocerca volvulus* cDNA encoding a low-molecular-weight antigen uniquely recognized by onchocerciasis patient sera. Mol Biochem Parasitol 1990;39:135–46. 1689459 10.1016/0166-6851(90)90016-f

[26] Ramachandran CP Improved immunodiagnostic tests to monitor onchocerciasis control programmes—a multicenter effort. Parasitol Today 1993;9(3):76–79. 10.1016/0169-4758(93)90204-s 15463714

[27] Chandrashekar R, Ogunrinade AF, Weil GJ Use of recombinant *Onchocerca volvulus* antigens for diagnosis and surveillance of human onchocerciasis. Trop Med Int Health 1996;1(5):575–80. 8911441 10.1111/j.1365-3156.1996.tb00082.x

[28] Lustigman S, Brotman B, Huima T et al Characterization of an *Onchocerca volvulus* cDNA clone encoding a genus specific antigen present in infective larvae and adult worms. Mol Biochem Parasitol 1991;45:65–76. 2052041 10.1016/0166-6851(91)90028-5

[29] Bradley JE, Trenholme KR, Gillespie AJ et al A sensitive serodiagnostic test for onchocerciasis using a cocktail of recombinant antigens. Am J Trop Med Hyg 1993;48(2):198–204. 8447523 10.4269/ajtmh.1993.48.198

[30] Bradley JE, Helm R, Lahaise M et al cDNA clones of *Onchocerca volvulus* low molecular weight antigens provide immunologically specific diagnostic probes. Mol Biochem Parasitol 1991;46(2):219–27. 1922197 10.1016/0166-6851(91)90046-9

[31] Mpagi JL, Buttner DW, Tischendorf FW et al Use of the recombinant *Onchocerca volvulus* protein Ov20/OvS1 for the immunodiagnostic differentiation between onchocerciasis and mansonelliasis and for the characterization of hyperreactive onchocerciasis (Sowda). Trop Med Int Health 2000;5(12):891–7. 11169279 10.1046/j.1365-3156.2000.00655.x

[32] Park J, Dickerson TJ, Janda KD Major sperm protein as a diagnostic antigen for onchocerciasis. Bioorg Med Chem 2008;16(15):7206–9. 18632276 10.1016/j.bmc.2008.06.038

[33] Lucius R, Kern A, Seeber F et al Specific and sensitive IgG4 immunodiagnosis of onchocerciasis with a recombinant 33 kD *Onchocerca volvulus* protein (Ov33). Tropical Medicine & Parasitology 1992;43(3):139–45. 1281926

[34] Tume CB, Ngu JL, McKerrow JL et al Characterization of a recombinant *Onchocerca volvulus* antigen (Ov33) produced in yeast. Am J Trop Med Hyg 1997;57(5):626–33. 9392607

[35] Lobos E, Weiss N, Karam M et al An immunogenic *Onchocerca volvulus* antigen: a specific and early marker of infection. Science 1991;251:1603–05. 2011741 10.1126/science.2011741

[36] Eberhard ML, Dickerson JW, Tsang VC et al *Onchocerca volvulus*: parasitologic and serologic responses in experimentally infected chimpanzees and mangabey monkeys. Exp Parasitol 1995;80(3):454–62. 7729480 10.1006/expr.1995.1057

[37] Ogunrinade AF, Chandrashekar R, Eberhard ML et al Preliminary evaluation of recombinant *Onchocerca volvulus* antigens for serodiagnosis of onchocerciasis. J Clin Microbiol 1993;31:1741–5. 8349749 10.1128/jcm.31.7.1741-1745.1993 PMC265624

[38] Weil GJ, Ogunrinade AF, Chandrashekar R et al IgG subclass antibody serology for onchocerciasis. J Infect Dis 1990;161:549–54. 2179425 10.1093/infdis/161.3.549

[39] Ogunrinade AF, Chandrashekar R, Weil GJ et al Use of a recombinant antigen (Oc3.6 cDNA) for the serological diagnosis of onchocerciasis in exposed Nigerian children. J Trop Pediatr 1992;38:103–105. 1507301 10.1093/tropej/38.3.103

[40] Ottesen EA, Skvaril F, Tripathy SP et al Prominence of IgG4 in the IgG antibody response to human filariasis. J Immunol 1985;134(4):2707–12. 2579154

[41] Lighaam LC, Rispens T The immunobiology of immunoglobulin G4. Semin Liver Dis 2016;36(3):200–15. 27466791 10.1055/s-0036-1584322

[42] Weil GJ, Steel C, Liftis F et al A rapid-format antibody card test for diagnosis of onchocerciasis. J Infect Dis 2000;182(6):1796–9. 11069258 10.1086/317629

[43] Golden A, Steel C, Yokobe L et al Extended result reading window in lateral flow tests detecting exposure to *Onchocerca volvulus*: a new technology to improve epidemiological surveillance tools. PLoS One 2013;8(7):e69231. 23935960 10.1371/journal.pone.0069231 PMC3720650

[44] Steel C, Golden A, Stevens E et al Rapid point-of-contact tool for mapping and integrated surveillance of *Wuchereria bancrofti* and *Onchocerca volvulus* infection. Clin Vaccine Immunol 2015;22(8):896–901. 26018537 10.1128/CVI.00227-15 PMC4519720

[45] PATH Quality assurance program materials for onchocerciasis and lymphatic filariasis tests. http://sites.path.org/dx/ntd/training-and-qaqc-materials/ [accessed 27 April 2017).

[46] Golden A, Stevens EJ, Yokobe L et al A recombinant positive control for serology diagnostic tests supporting elimination of *Onchocerca volvulus*. PLoS Negl Trop Dis 2016;10(1):e0004292. 26745374 10.1371/journal.pntd.0004292 PMC4706346

[47] Dieye Y, Storey HL, Barrett KL et al Feasibility of utilizing the SD BIOLINE Onchocerciasis IgG4 rapid test in onchocerciasis surveillance in Senegal. PLoS Negl Trop Dis 2017;11(10):e0005884. 28972982 10.1371/journal.pntd.0005884 PMC5640270

[48] Trees AJ, McCall PJ, Davies JB On the possibility of bovine *Onchocerca* species infecting *Simulium damnosum* s.l. in the forest zone of Sierra Leone. I. Parasitological aspects. Ann Trop Med Parasitol 1989;83(6):595–601. 2619374 10.1080/00034983.1989.11812393

[49] Katholi CR, Toe L, Merriweather A et al Determining the prevalence of *Onchocerca volvulus* infection in vector populations by polymerase chain reaction screening of pools of black flies. J Infect Dis 1995;172(5):1414–17. 7594692 10.1093/infdis/172.5.1414

[50] Yameogo L, Toe L, Hougard JM et al Pool screen polymerase chain reaction for estimating the prevalence of *Onchocerca volvulus* infection in *Simulium damnosum* sensu *lato*: results of a field trial in an area subject to successful vector control. Am J Trop Med Hyg 1999;60(1):124–8. 9988335 10.4269/ajtmh.1999.60.124

[51] Rodríguez-Pérez MA, Danis-Lozano R, Rodríguez MH et al Detection of *Onchocerca volvulus* infection in *Simulium ochraceum* sensu *lato*: comparison of a PCR assay and fly dissection in a Mexican hypoendemic community. Parasitology 1999;119(6):613–19. 10633923 10.1017/s0031182099005107

[52] Rodriguez-Perez MA, Fernandez-Santos NA, Orozco-Algarra ME et al Elimination of onchocerciasis from Mexico. PLoS Negl Trop Dis 2015;9(7):e0003922. 26161558 10.1371/journal.pntd.0003922 PMC4498594

[53] Richards F Jr, Rizzo N, Diaz Espinoza CE et al One hundred years after its discovery in Guatemala by Rodolfo Robles, *Onchocerca volvulus* transmission has been eliminated from the central endemic zone. Am J Trop Med Hyg 2015;93(6):1295–304. 26503275 10.4269/ajtmh.15-0364 PMC4674249

[54] Zarroug IM, Hashim K, ElMubark WA et al The first confirmed elimination of an onchocerciasis focus in Africa: Abu Hamed, Sudan. Am J Trop Med Hyg 2016;27:1037–40. 10.4269/ajtmh.16-0274 PMC5094213 27352878

[55] Rodríguez-Pérez MA, Adeleke MA, Burkett-Cadena ND et al Development of a novel trap for the collection of black flies of the *Simulium ochraceum* complex. PLoS One 2013;8(10):e76814. 24116169 10.1371/journal.pone.0076814 PMC3792067

[56] Rodríguez-Pérez MA, Adeleke MA, Rodríguez-Luna IC et al Evaluation of a community-based trapping program to collect *Simulium ochraceum sensu lato* for verification of onchocerciasis elimination. PLoS Negl Trop Dis 2014;8:e3249. 25340517 10.1371/journal.pntd.0003249 PMC4207651

[57] Sauerbrey M; The Onchocerciasis Elimination Program for the Americas (OEPA). Ann Trop Med Parasitol 2008;102(Suppl. 1):25–9. 18718151 10.1179/136485908X337454

[58] Garms R, Walsh JF, Davies JB Studies on the reinvasion of the Onchocerciasis Control Programme in the Volta River Basin by *Simulium damnosum* s.l. with emphasis on the south-western areas. Tropenmed Parasitol 1979;30(3):345–62. 575581

[59] Lont YL, Coffeng LE, de Vlas SJ et al Modelling anti-Ov16 IgG4 antibody prevalence as an indicator for evaluation and decision making in onchocerciasis elimination programmes. PLoS Negl Trop Dis 2017;11(1):e0005314. 28114304 10.1371/journal.pntd.0005314 PMC5289624

[60] Walker M, Specht S, Churcher TS et al Therapeutic efficacy and macrofilaricidal activity of doxycycline for the treatment of river blindness. Clin Infect Dis 2015;60(8):1199–207. 25537873 10.1093/cid/ciu1152 PMC4370165

[61] Denery JR, Nunes AA, Hixon MS et al Metabolomics-based discovery of diagnostic biomarkers for onchocerciasis. PLoS Negl Trop Dis 2010;4(10):pii: e834. 10.1371/journal.pntd.0000834 PMC2950146 20957145

[62] Globisch D, Moreno AY, Hixon MS et al *Onchocerca volvulus*-neurotransmitter tyramine is a biomarker for river blindness. Proc Natl Acad Sci USA 2013;110(11):4218–23. 23440222 10.1073/pnas.1221969110 PMC3600455

[63] Tritten L, Burkman E, Moorhead A et al Detection of circulating parasite-derived microRNAs in filarial infections. PLoS Negl Trop Dis 2014;8(7):e2971. 25033073 10.1371/journal.pntd.0002971 PMC4102413

